# Plaque Rupture-Induced Myocardial Infarction and Mechanical Circulatory Support in Alpha-Gal Allergy

**DOI:** 10.1155/2020/5282843

**Published:** 2020-02-17

**Authors:** Sohab S. Radwan, Gauravpal Gill, Amre Ghazzal, Awais Malik, Christopher Barnett

**Affiliations:** ^1^MedStar Washington Hospital Center, Washington, DC, USA; ^2^MedStar Georgetown University Hospital, Washington, DC, USA

## Abstract

Alpha-gal (AG) allergy is an IgE-mediated allergic reaction to galactose-alpha-1,3-galactose found in mammalian meat. Heparin, being derived from porcine intestinal tissue, may have a degree of cross-reactivity with AG antigen and thus place patients at risk for allergic and even anaphylactic reactions. This is especially important in patients with myocardial infarction (MI) and mechanical circulatory support, such as a left ventricular assist device (LVAD), since anticoagulation is immediately required. Therefore, individualized assessment and preoperative planning is needed regarding the use of heparin vs. nonheparinoid products in such a population.

## 1. Introduction

AG allergy, also known as AG syndrome or red meat allergy, is an IgE-mediated allergic reaction to galactose-alpha-1,3-galactose carbohydrate found in mammalian meat such as pork and beef. It usually occurs following a tick bite, with the lone star tick being the most predominant cause in the US [1]. The prevalence of AG allergy is steadily increasing with an estimated 10% of certain populations, especially in southeastern US, reported to have elevated antibody titers against AG antigen [2]. The severity of the reaction varies, but it usually manifests as a late yet powerful anaphylactic reaction 3-6 hours after ingestion, rather than a simple allergic one, and is therefore important to recognize [3]. Heparin products, either unfractionated or low-molecular weight, are derived from porcine intestinal tissue. Although data on the degree of heparin cross-reactivity with AG antigen is still lacking, it is highly likely that such patients will react to heparin [4]. Intolerance to heparin products poses challenges for anticoagulation in patients with MI and postoperatively, in those with mechanical circulatory support devices such as LVAD.

## 2. Case Presentation

A 56-year-old male with a past medical history of coronary artery disease (status post percutaneous coronary intervention (PCI) to the distal left anterior descending artery (LAD) in 2011), ischemic cardiomyopathy (ejection fraction of 20-25%), type 2 diabetes mellitus, hypertension, hyperlipidemia, and AG allergy presented with chest pain and dyspnea on exertion of one-day duration. On initial presentation, he was hemodynamically stable. Electrocardiogram revealed 3 mm ST segment elevation in leads V2-V4 and reciprocal 1 mm ST segment depression in leads I and aVL, consistent with a diagnosis of anterior wall ST segment elevation MI. The patient was taken immediately to the catheterization lab, where he underwent left heart catheterization, which revealed 50% stenosis of the proximal LAD, 100% occluded mid LAD with thrombus in place, and 30% in-stent restenosis in the distal LAD. He underwent PCI with successful drug-eluting stent placement in the mid LAD. He was anticoagulated with bivalirudin given his AG allergy. His hospital course was complicated by cardiogenic shock approximately 48 hours following PCI requiring intravenous diuresis and inotrope support. His hemodynamics subsequently deteriorated, and a refractory requirement for progressively increasing doses of inotropes leads to evaluation for advanced heart failure therapies. He underwent an exhaustive evaluation and planning for LVAD placement with periprocedural anticoagulation using bivalirudin. He was eventually discharged to a cardiac rehabilitation center. The patient is currently doing well and follows up periodically in the advanced heart failure clinic at our hospital.

## 3. Discussion

This article describes a case of anterior wall ST segment elevation MI leading to shock requiring inotrope therapy and eventually mechanical circulatory support. A history of ischemic cardiomyopathy with poor functional reserve at an ejection fraction of 20-25% at baseline, along with new acute coronary plaque rupture, leads to cardiogenic shock in our patient which did not resolve with revascularization and diuresis. An important consideration on the differential was a separate entity of acute coronary syndrome, previously described in the literature, called Kounis syndrome. Also known as allergic angina or allergic acute coronary syndrome, Kounis syndrome is usually precipitated by an acute allergic reaction triggering the release of several inflammatory mediators [5]. Pathogenesis involves mast cell activation and degranulation, which can ultimately lead to coronary artery vasospasm and atheromatous plaque erosion, rupture, and thrombus formation [5, 6]. It is diagnosed infrequently; however, it is common in individuals with an allergic background [5]. Despite having a significant allergic history, anaphylaxis is a less likely etiology for shock in this patient without exposure to known allergens and stable hemodynamics on presentation. Therefore, while managing this patient, serum tryptase levels, which reflect mast cell burden and help in determining the presence of anaphylaxis, were not measured [7].

There are no available standardized desensitization protocols that could have facilitated the use of heparin in this patient. Moreover, the amount of heparin required to trigger an allergic reaction is unclear, since a nonstandardized amount of AG antigen is present in each lot of heparin [4]. However, there have been reported cases of patients with AG allergy who received heparin products during cardiac surgery after being preoperatively treated with steroids and antihistamines, with acceptable outcomes. Interestingly, heparin has also been shown to induce anaphylaxis in individuals without AG allergy [8]. This mainly occurs as part of the heparin-induced thrombocytopenia syndrome as well as through the contact system-activating effects of unknown contaminants within heparin lots [9]. Given the risk for cross-reactivity between heparin and AG antigen, proposed alternative options for anticoagulation include bivalirudin and direct oral anticoagulants, specifically apixaban or rivaroxaban [10]. However, the lack of experience with those drugs and varied duration of action in this setting as compared to heparin presents a challenge as well.

Furthermore, stents themselves may act as antigenic stimuli and induce hypersensitivity reactions [11]. Culprit components include the polymer coating, metallic platform, and the released drugs, which are thought to result in chronic and repetitive hypersensitivity irritation to the coronary intima [11]. This eventually results in attracting several proteins, mainly fibrinogen and complement, leading to complement system activation [11]. As such, this constitutes a variant of allergic acute coronary syndrome, manifesting as stent thrombosis, which is unlikely in our patient given the sequence of events. The current emphasis in research revolves around the use of biodegradable stent technology in an effort to reduce the trigger for persistent inflammation within the coronary intima [12]. With such technology, all stent components (drug, polymer, or scaffold) are absorbed and none is left behind [13]. Although the evidence on such technology is promising, it is still limited [14].

## 4. Conclusion

Further research regarding AG cross-reactivity with heparin in acute coronary syndrome as well as perioperative anticoagulation alternatives in mechanical circulatory support is necessary. Moreover, hypersensitivity reactions to implanted materials within stent structures must be emphasized on. Lastly, establishing standardized heparin desensitization protocols in patients with AG allergy is crucial.
